# Involvement of tumor suppressors PTEN and p53 in the formation of multiple subtypes of liposarcoma

**DOI:** 10.1038/cdd.2015.27

**Published:** 2015-03-27

**Authors:** A M Puzio-Kuter, S V Laddha, M Castillo-Martin, Y Sun, C Cordon-Cardo, C S Chan, A J Levine

**Affiliations:** 1 Rutgers Cancer Institute of New Jersey, New Brunswick, New Jersey, USA; 2 Icahn School of Medicine at Mount Sinai, Mount Sinai School of Medicine, New York, USA; 3 Department of Medicine, Rutgers Robert Wood Johnson Medical School, New Jersey, USA; 4 Institute for Advanced Study, Princeton, New Jersey, USA

## Abstract

Liposarcoma (LPS) is a type of soft tissue sarcoma that mostly occurs in adults, and in humans is characterized by amplifications of *MDM2* and *CDK4*. The molecular pathogenesis of this malignancy is still poorly understood and, therefore, we developed a mouse model with conditional inactivation of PTEN and p53 to investigate these pathways in the progression of the disease. We show that deletion of these two tumor suppressors cooperate in the formation of multiple subtypes of LPS (from well-differentiated LPS to pleomorphic LPS). In addition, progression of the tumors is further characterized by the expression of D cyclins and CDK4/6, which allow for continued cell division. Microarray analysis also revealed novel genes that are differentially expressed between different subtypes of LPS, which could aid in understanding the disease and to unravel potential new therapeutic targets.

Liposarcoma (LPS) is a neoplasm that arises from adipose tissue and accounts for ~20% of the soft tissue and bone sarcoma that develop in the United States.^1^ There are four main histological subtypes of LPSs that are classified morphologically and consist of well-differentiated LPS (WDLPS), dedifferentiated LPS (DDLPS), myxoid/round cell LPS (MLPS) and pleomorphic LPS (PLPS). The subtypes differ in terms of biological behavior and metastatic potential, with WDLPS having a low recurrence rate and a lower tendency to metastasize than MLPS or PLPS.^2^ The treatment options are mainly surgery and radiation with chemotherapy for systemic disease. Though depending upon the subtype there is variation in terms of chemotherapy sensitivity, and few options exist for aggressive local or metastatic disease.^3^

Chromosomal amplifications of the 12q13–15 region, which include the *MDM2*, *CDK4* and *HMGA2* genes, among others, are a characteristic feature of LPSs.^4, 5, 6^ *MDM2* amplification occurs in several cancers and is thus of importance in tumorigenesis.^7^ MDM2, which is a negative regulator of p53, blocks p53-dependent transcription and recruitment of transcription coactivators, by binding within its *N*-terminal hydrophobic pocket. MDM2 also has been reported to have p53-independent functions that control proliferation, apoptosis, epithelial-to-mesenchymal transition and the initiation of adipocyte differentiation.^7, 8^ In WDLPS, amplification of *MDM2* and p53 mutations seem to be mutually exclusive and p53 mutations have been associated with the dedifferentiation process from WDLPS to DDLPS. Also the location of LPSs in the body appears to be an important factor. In retroperitoneal WDLPS/DDLPS tumors *MDM2* amplifications and p53 mutations were mutually exclusive but in non-retroperitoneal DDLPS tumors p53 mutations occur in the presence of *MDM2* amplifications.^9, 10^ The latter implicates a role for mutant p53 in the dedifferentiation process. Interestingly, some of the most common cancers found in p53 heterozygote Li-Fraumeni patients are soft tissue sarcomas such as LPSs, fibrosarcomas, and rhabdomyosarcomas.^11, 12^

In addition to the p53 pathway, the PI3K-AKT pathway is commonly dysregulated in human cancer. Its downstream effects include protein synthesis, genetic stability, cellular proliferation and survival. Mutations in PTEN, which normally regulate the PI3K-AKT pathway, have been found in multiple lipomas (benign adipocytic neoplasms)^13^ and AKT activation has been found in human LPSs.^14^ Therefore, the role that this pathway has in adipocyte transformation predicts it as a potential target pathway for therapeutics.

In this study, we sought to understand the combined role of *p53* and *Pten* deletions in a mouse model of LPS. Accumulating evidence from zebrafish and xenograph models have begun to address their combined importance in this tumor type.^15, 16^ Using conditional inactivation of tumor suppressors p53 and PTEN in adipose tissue, we show that deletion of *p53* and *Pten* together results in tumor formation in ~85% of mice with tumors representing all four major subtypes of LPS observed in humans. A gene expression microarray analysis of these tumor types indicates many differences in gene expression and unravels a new possible method of diagnosis of these tumors. This mouse model also demonstrates a role for the MDM2 protein in the formation of some LPS cell types even in the absence of the p53 functions. Once p53 and PTEN functions are eliminated, the next step in the transformation pathway of LPSs is the enhanced production of some D cyclins and CDK4/6, leading to continual signaling for cell division. Interestingly this is the same pathway observed when p53 knockout mice produce T-cell lymphomas.^17^

## Results

### High penetrance of LPS formation initiated by PTEN and p53 deficiency

**Figure 1:**
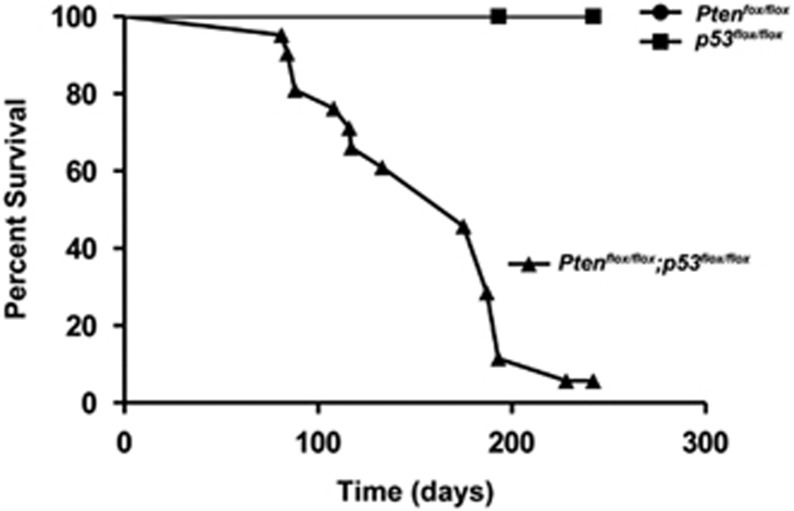
Survival curve of *p53*^*flox/flox*^, *Pten*^*flox/flox*^ and *Pten*^*flox/flox*^*; p53*^*flox/flox*^ mice. Adenovirus-cre injection into adipose tissue of male and female *p53*-floxed, and *Pten-*floxed allele mice in combination leads to liposarcoma at the indicated time points. *p53*^*flox/flox*^ (*n*= 8), *Pten*^*flox/flox*^ (*n*= 8) and *Pten*^*flox/flox*^*; p53*^*flox/flox*^ (*n*= 22)

Accumulating evidence points to important roles for the p53 and PI3K/PTEN pathway in the development of LPSs. In this study, we examined the consequences of deletion of *Pten* and *p53* in adipose tissue of mice. Adenovirus-cre was either injected into the adipose tissue surrounding the ovary (gonadal fat pad) or surrounding the testes resulting in either deletion of exons 2–10 in *p53*^*flox/flox*^ mice,^18^ or exon 5 in the *Pten*^*flox/flox*^ mice.^19^ Injection of adenovirus-cre into adipose tissue of either *p53*^*flox/flox*^*, Pten*^*+/+*^ mice or *p53*^*+/+*^*, Pten*^*flox/flox*^ mice led to no tumor formation, whereas injection of *p53*^*flox/flox*^*, Pten*^*flox/flox*^ mice led to >85% of mice with tumor formation (Figure 1). Tumors in the *p53*^*flox/flox*^*, Pten*^*flox/flox*^ mice were detected by palpation as early as 81 days post injection and were 50% penetrant at 153 days. Thus, deletion of both alleles of *p53* and *Pten*, in combination, results in highly penetrant LPS development in this mouse model.

### Development of multiple subtypes of LPS in *p53^flox/flox^; Pten^flox/flox^* mice

**Figure 2:**
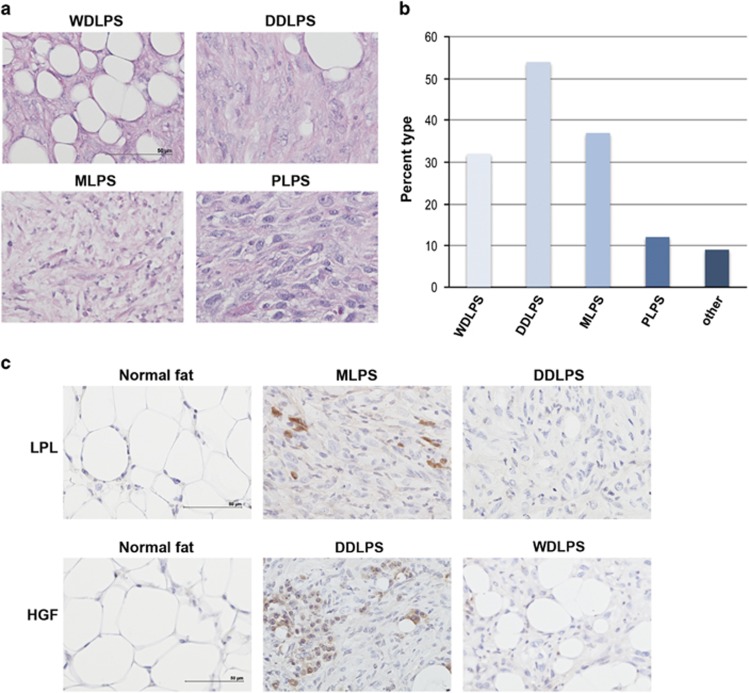
Classification of liposarcomas generated in the *Pten*^*flox/flox*^*; p53*^*flox/flox*^ mouse model. (**a**) Representative hematoxylin and eosin (H&E) of the four major subtypes of liposarcoma identified; well-differentiated liposarcoma (WDPLS), dedifferentiated liposarcoma (DDLPS), myxoid liposarcoma (MPLS) and pleomorphic liposarcoma (PLPS). (**b**) Bar diagram graph represents the percentage of the each of the subtypes of liposarcoma. A small percentage of other subtypes of sarcomas were identified, including rhabdomyosarcoma and osteosarcoma. (**c**) Different subtypes of liposarcoma are characterized by a unique lipocytic phenotype. Lipoprotein lipase (LPL) is an adipocytic marker and stains more mature adipocytic sarcomas such as WDLPS and MLPS. Hepatocyte growth factor (HGF) is a mesenchymal stem cell marker and stains immature liposarcomas such as DDLPS and PLPS. Scale bar corresponds to 50 *μ*m

Histological analysis of the LPSs, which formed in the *p53*^*flox/lox*^*; Pten*^*flox/flox*^ mice, showed that all four main subtypes, WDPLS, DDPLS, MLPS and PLPS, were represented (Figure 2a). Interestingly, within the same tumor one or two different subtypes could be found. Evidence points to mesenchymal stem cells (MSCs) being the target cell for the development of sarcomas and LPSs from which multiple types of these tumors are possible.^20^ Approximately, 80% of the tumors examined had a dedifferentiated subtype component, whereas the pleomorphic subtype represented the least number of cases (Figure 2b). Marker analysis to verify the histologically identified subtypes showed that WDLPS and MLPS, which have been classified as being more mature adipocytic sarcomas, were characterized by high expression of adipocytic markers (LPL) when compared with DDLPS and PLPS (Figure 2c, upper panels). In the contrary, DDLPS and PLPS subtypes, which are classified as being more immature LPSs, display stronger and more diffuse expression of MSC markers (HGF) than WDLPS and MLPS (Figure 2c, lower panels, and data not shown).^21^

### Expression of cell cycle regulation genes in multiple subtypes of *p53^flox/flox^; Pten^flox/flox^* LPS tumors

**Figure 3:**
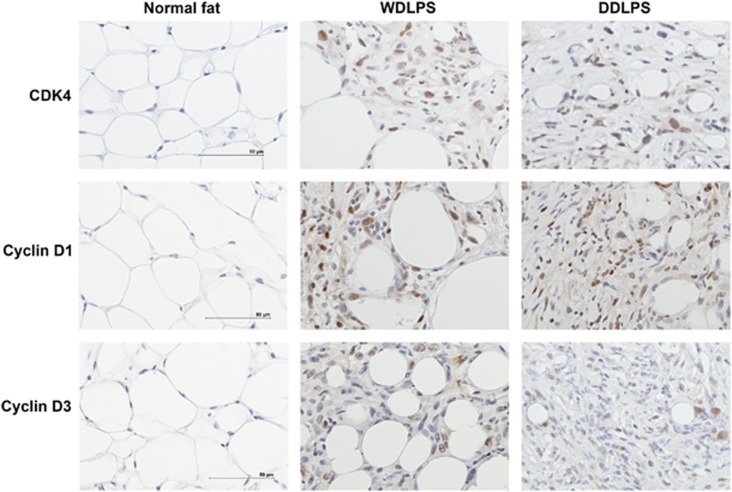
Immunohistochemical analysis of cell cycle markers in WDLPS and DDLPS subtypes. There is a deregulation in the G1–S checkpoint with the upregulation of CDK4, Cyclin D1 and Cyclin D3. Scale bar corresponds to 50 *μ*m

Cell cycle regulation in human cancer is often deregulated resulting in unscheduled proliferation, genomic instability and chromosomal instability leading to aneuplody. Cyclin-dependent kinases (CDK) and cyclins are the main driving forces of cell cycle regulation and they are in turn regulated by p53 and PTEN. Therefore, we wanted to investigate some of the molecular consequences of p53 and PTEN loss on these proteins in LPS. Here we found that there is an aberration in the G1–S checkpoint with the upregulation of CDK4, CDK6, Cyclin D1 and Cyclin D3 in all four of the subtypes of LPS (WDLPS, DDLPS, MLPS and PLPS) (Figure 3 and data not shown). The available evidence suggests that Cyclin D1 and Cyclin D3 can be regulated by PTEN,^22, 23^ however, this is the first time it has been shown that they are overexpressed in the absence of both p53 and PTEN in a mouse model of LPS. These results suggest that alterations of this pathway are essential for the oncogenic process of the different subtypes of LPS.

### MDM2 expression in subtypes of mouse LPSs

**Figure 4:**
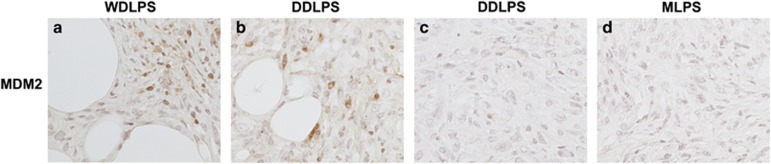
Immunohistochemical analysis of MDM2 in WDLPS, DDLPS and MPLS subtypes. MDM2 protein is expressed at very high levels in WDLPS and DDLPS sections in areas with lipoblasts (**a** and **b**) and absent in DDLPS sections lacking lipoblasts (**c**) and MLPS (**d**)

Most LPSs have amplifications of the MDM2 gene with a smaller number of LPSs having loss or mutation of the TP53 gene. It is assumed that MDM2 amplification would be similar to a p53 loss or mutation on affecting downstream pathways. However, with increasing evidence that MDM2 has p53-independent roles we, therefore, wanted to investigate the status of MDM2 in tissue sections of *p53*^*flox/flox*^*; Pten*^*flox/flox*^ LPS tumors.^7, 8^ We investigated MDM2 RNA levels by real-time PCR (Taqman) in normal *versus* tumor tissue but did not see an appreciable difference between normal fat and tumors (1.5- to twofold, data not shown) as seen in human LPS samples.^24^ Therefore, we examined the protein expression of MDM2 by immunohistochemistry. Interestingly, MDM2 protein is expressed at very high levels in WDLPS and DDLPS sections in areas with clear lipoblasts (Figure 4, panels a and b) and absent from DDLPS sections lacking lipoblasts and MLPS (Figure 4, panels c and d). Similarly, this has been seen in the case of CDK4 where it is not amplified in the well-differentiated component of a tumor and amplified in the dedifferentiated component.^25^

Given MDM2's p53-independent role in initiation of adipocyte differentiation,^8^ MDM2 expressing areas might be undergoing a dedifferentiation process that is controlled by MDM2. We performed IHC analysis in serial sections using MDM2 and C/EBP delta, which has been shown to be important for the dedifferentiation process. Interestingly, MDM2 and C/EBP delta showed similar patterns of expression around lipoblasts (Supplementary Figure 1), which allows for a potential explanation for the restricted MDM2 expression pattern and dedifferentiation from WDLPS to DDLPS.

### Bioinformatics analysis of differentially expressed genes in two of the biological types of LPS

**Figure 5:**
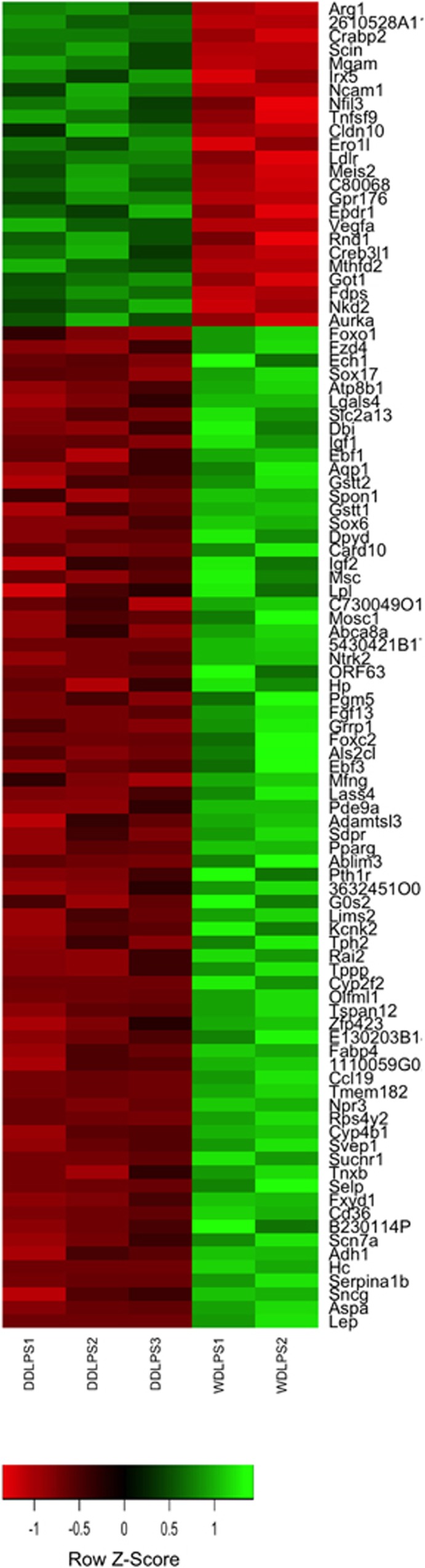
Heat Map of differentially expressed genes between WDPLS and DDLPS. The heat map was generated using genes with *P*-value ≤0.01 (uncorrected) and fold change >3

To understand the possible progression of WDLPS to DDLPS, we analyzed gene expression profiles by microarray to identify pathways and genes that might be differentially expressed. This would aid in understanding the molecular consequences for the loss of p53 and PTEN pathways in different subtypes of LPS. The differentially expressed genes between WDPLS and DDLPS are shown in a Heat Map (Figure 5, expression data for all samples in Supplementary file 2). While this list of genes is not statistically significant due to limited number of samples and multiple hypotheses testing, it suggests candidate genes in differentiation between the two subtypes as well as potential new markers of tumorigenesis and therapeutic targets. Table 1 shows the upregulated genes in each subtype compared with the other subtype that have a greater than threefold change in expression and *P* value <0.01 (genes that have a twofold change but of particular importance are marked in the table). Many of the genes that are of interest, in distinction of the two subtypes, relate to the control of lipid, fatty acid, amino acid and carbohydrate metabolism (*Fabp4, Lpl, Pgm5, Ldlr, Got1, Ech1),* insulin-like growth factor pathway (*Igf2, Igf1,Foxo1*), transcription factors (*Foxc2, Sox6, Pparg, Irx5, Meis2, Ebf1, Sox17*), obesity-related genes (*Lep, Ntrk2),* Wnt signaling (*Nkd2*, *Fzd4*) or genes related to cell proliferation and apoptosis (Vegfa*, G0s2*). Two genes that are p53 regulated or regulate p53, *Got1* and *Aurka,* respectively, might help to explain targets that are of interest in the formation of LPSs in the absence of p53. These data suggest potentially novel genes that can be used to distinguish between WDPLS and DDLPS subtypes and can also provide insight into LPS pathogenesis and progression.

## Disscussion

High-level amplification of the *MDM2* and *CDK4* genes have become markers for studying human LPSs, implicating deregulation of both the p53 pathway and cell cycle regulation as necessary aspects for tumor progression. However, researchers have also seen that other points in the p53 pathway can be deregulated such as p53 itself with or without *MDM2* amplification.^9, 10^ In addition, another widely mutated gene in cancer, *PTEN*, a regulator of the PI3K-AKT pathway, has been found in multiple lipomas (benign adipocytic neoplasms)^13^ and AKT activation has been found in human LPSs.^14^ This finding is of significance owing to the many drugs that have been found to target the PI3K-AKT-mTOR pathway and the lack of drugs currently effective for the treatment of LPSs or that are still in the clinical trial period.^26^ In this study, we developed a mouse model of LPS that demonstrates the combined effect of p53 and PTEN deletion in the generation of these tumors and its potential utility in the development of novel therapeutics.

The interaction of the p53 and PI3K/AKT/PTEN pathways is well documented. Likewise, combinatorial deletion or inactivation of members from each pathway is also very common. In this mouse model, we observe that when either alleles of *p53* or *Pten* were deleted individually it did not result in any tumor formation but in combination several different subtypes of LPS were formed. It could be that inactivation of both pathways in a stem cell or progenitor type cell could lead to the formation of the different subtypes of LPS (WDLPS, DDLPS, MLPS and PLPS). Two previous studies, have suggested this possibility as well as a way for the progression of tumorigenesis.^21, 27^

In addition, one of the mechanisms that could be the driving force for tumor progression, in the absence of p53 and PTEN, is cell cycle regulation. In this model of LPS, the loss of p53 and PTEN promotes the abnormal expression of D cyclins and CDK4/6. CDKs are bound by regulatory subunits called cyclins that are in turn made at certain times during the cell cycle and regulate enzymatic activity. Mutations that deregulate these CDK-cyclin complexes lead to continued proliferation or inappropriate re-entry into the cell cycle. In this model, altered expression of these cell cycle regulation proteins could be one of the driving forces of uncontrolled growth and LPS tumorigenesis. Specifically, the increased expression of the G_1_–S phase regulators CDK4 and cyclin E would serve to enhance the G_1_–S transition.

It is of some interest that p53 knockout mice develop thymic lymphomas. These tumors select for a common series of mutations in a specific order. After the gatekeeper mutation of *p53*, then *Pten* deletions are commonly selected for and this is followed by *Cyclin D1, D2, D3* and *Cdk-6* amplifications and overexpression. The LPSs described here have demonstrated an identical series of events, suggesting a common pathway to diverse cancers following a *p53* mutation.

Other mechanisms involved in this tumor model could be regulating the dedifferentiation of tumor subtypes. MDM2 has been shown to exert a p53-independent role on the initiation of adipocyte differentiation through controlling CREB-dependent transactivation, as well as the determination of cell fate of MSCs, another p53-independent function. Adipocytes, osteoblasts, myocytes and chondrocytes all differentiate from MSCs.^8^ In the current study, MDM2 expression was found in areas of lipocytes in both WDLPS and DDLPS *p53*^*flox/flox*^*; Pten*^*flox/flox*^ tumors. Therefore, in this model MDM2 might be directing these cells toward a more dedifferentiated cell type, a hypothesis that still needs to be further tested. Another potential way that MDM2 might direct cells toward a dedifferentiated phenotype is through epigenetic reprogramming. MDM2-dependent degradation of RB increased levels and activity of DNA methyltransferase DNMT3A^28^ and thus silencing of tumor suppressor genes. Similarly, MDM2 interacts with the histone methyltransferase SUV39H1 and can in certain instances lead to repression of p53 target genes^29, 30^ and potentially epigenetic reprogramming.^31^

To begin to understand how WDLPS and DDLPS differ molecularly in this mouse model in the presence of p53 and PTEN deletions we ran gene expression microarray analysis to compare the two different LPS tumor types. Our investigation would hopefully lead to the discovery of novel deregulated pathways, potential new therapeutic targets and pathways involved in the dedifferentiation process of the subtypes. Some deregulated genes and pathways were similar to a previous human study of WDLPS and DDLPS^32^ (lipid, fatty acid and carbohydrate metabolism: *Fabp4, Lpl,* transcription factors: *Fox and Sox* family members), however some novel ones were also found (insulin-like growth factor pathway: *Igf1, Igf2*). The expression of the insulin-like growth factor pathway genes is most likely due to the interaction of both the p53 and PTEN pathways being deregulated. The insulin-like growth factor pathway has been an attractive target for therapies and its importance in LPS tumorigenesis is becoming more evident. Therefore, it is interesting that IGF2 has recently been identified as a potential therapeutic target and in esophageal cancers has been shown to be suppressive toward tumor growth and metastasis.^33^ Another pathway, Wnt signaling, which is highly involved in development and adipogenesis could be another key pathway that should be investigated in LPS.^34^

We also have some clues as to transcription factors that might be working to induce dedifferentiation through epigenetic mechanisms. SOX6 is an important developmental gene as well as involved in differentiation. It is also known to suppress cyclin D1 activities by interacting with beta-catenin and HDAC1, thus, leading to a decrease in acetylated H3 and H4 at the cyclin D1 promoter.^35^ SOX6 is misexpressed in the tumor subtypes though it is unknown if it works in conjunction with MDM2 to promote these processes.

Genes that are p53 regulated or regulate p53 might aid in the investigation of mechanisms responsible for LPS formation when p53 and PTEN are deleted in adipose tissue. *Got1* and *Aurka* were found to be upregulated in DDLPS. Through Got1, p53 might be a novel regulator of glucose production and inhibit the use of glucose in pathways that promote tumorigenesis.^36^ Likewise, Aurka has been shown to be important not only in the p53 pathway but also in the Pten/Akt pathway. Specifically, Aurka regulates ESC pluripotency through phosphorylation-mediated inhibition of p53-directed gene expression.^37^ Inhibition of Aurka in the absence of p53 made cells more susceptible to mitotic arrest and slippage.^38^ Similarly, Aurka inhibition can downregulate Akt and promotes significant cell death.^39^

In summary, we have shown that combined deletion of p53 and PTEN leads to the formation of several subtypes of LPS in a mouse model of the disease. Molecular mechanisms involved in the progression include deregulation of cell cycle genes leading to uncontrolled cell proliferation as well as upregulation of MDM2, which might promote dedifferentiation and epigenetic reprogramming. Finally, gene expression microarray analysis has given us not previously known genes that can be further investigated in exploring these hypotheses.

## Materials and Methods

### Animal model

All animal study protocols were approved by the University of Medicine and Dentistry of New Jersey Institutional Animal Care and Use Committee review board. Conditional alleles for Pten (Pten^Tm1Hwu^) (Lesche *et al.*^19^) and p53 (Trp53^Tm1Brn^, Jonkers *et al.*^18^) mice were purchased from Jackson Laboratories, Bar Harbor, ME, USA. In all, 8–10-week old mice were used for injection of an adenovirus expressing cre recombinase (Ad5CMVCre, University of Iowa's Vector Core Facility (http://www.uiowa.edu/~gene) into adipose tissue. Concentrated virus (25 *μ*l; 4 X10^11^PFU/ml) was mixed with 20 *μ*l of Dulbecco's modified eagle medium (D-MEM). Mice were monitored for tumor development by palpitation at weekly intervals. Kaplan–Meier analysis was generated using GraphPad software.

### Histological analysis

Adipose tissue and tumor samples were harvested and fixed in 10% formalin overnight. The samples were embedded in paraffin and sectioned. The sections were subjected to hemotoxylin and eosin staining or immunohistochemical staining following the standard avidin-biotin protocol. The following antibodies were used: MDM2 (clone SMP14 Abcam, Cambridge, MA, USA), LPL (Abcam ab21356), HGF (Novus Biologicals NBP1-19182, Littleton, CO, USA), CDK4 (Abcam ab7955), CDK6 (Novus Biologicals NB100-91722), Cyclin D1 (Cell Signaling 2978, Danvers, MA, USA), Cyclin D3 (Cell Signaling 2936), C/EBP delta (antibodies-online, Atlanta, GA, USA; ABIN233849).

### Gene expression microarray

Total RNA was isolated from frozen tissue using TRIzol (Life Technologies) followed by clean-up with the Qiagen RNeasy kit and subjected to the 2100 Bioanalyzer from Agilent Technologies. cDNA samples were generated and labeled using Affymetrix 1-cycle expression kit (Affymetrix, Santa Clara, CA, USA) and hybridized to Affymetrix GeneChip Mouse Genome 430 2.0 Array using Affymetrix hybridization kit materials according to manufacturer's instruction. The raw Affymetrix. CEL files were imported into GeneSpring GX11 software (Silicon Genetics, Redwood City, CA, USA). Quality control was performed to remove bad-quality probes. Normalized expression values were calculated by the GC Robust Multi-array Average algorithm and subjected to mean transformation to collapsed all probe sets to respective genes in R. Collapsed gene expression values from all samples were used to find differentially expressed genes. The mean gene expression of each liposarcoma subtype was used to calculate the fold change between subtypes. We used gene expression fold change threshold of 3 and uncorrected *T*-test *P*-value of less than 0.01 to define a list of differentially expressed genes between the subtypes. Bonferroni correction was not used due to limited number of samples.

## Figures and Tables

**Table 1:** Upregulated genes, with a greater than threefold change in expression, in WDLPS and DDLPS as compared with each other

**Gene**	**Title**	**Fold change**
*Upregulated in well-differentiated liposarcoma*		
Lep	Leptin (murine obesity homolog)	151.98
Aspa	Aspartoacylase	58.72
Adh1	Alcohol dehydrogenase 1A (class I), alpha polypeptide	35.11
Cd36	CD36 molecule (thrombospondin receptor)	25.57
Sucnr1	Succinate receptor 1	21.78
Cyp4b1	Cytochrome P450, family 4, subfamily B, polypeptide 1	20.87
Fabp4	Fatty-acid binding protein 4, adipocyte	15.48
Zfp423	Zinc finger protein 423	13.77
Rai2	Retinoic-acid induced 2	11.79
G0s2	G0/G1 switch 2	10.03
Ablim3	Actin-binding LIM protein family, member 3	9.31
Pparg	Peroxisome proliferator-activated receptor gamma	9.16
Ebf3	Early B-cell factor 3	6.92
Foxc2	Forkhead box C2 (MFH-1, mesenchyme forkhead 1)	6.03
Fgf13	Fibroblast growth factor 13	5.77
Pgm5	Phosphoglucomutase 5	5.47
Ntrk2	Neurotrophic tyrosine kinase, receptor, type 2	5.12
Lpl*	Lipoprotein lipase	4.37
Igf2*	Insulin-like growth factor 2	4.18
Card10	Caspase recruitment domain family, member 10	4.09
Sox6	SRY (sex determining region Y)-box 6	4.05
Ebf1	Early B-cell factor 1	3.29
Igf1	Insulin-like growth factor 1	3.27
Sox17	SRY (sex determining region Y)-box 17	3.13
Ech1	Enoyl CoA hydratase 1	3.04
Fzd4^#^	Frizzled class receptor 4	2.47
Foxo1^#^	Forkhead box O1	2.01
		
*Upregulated in dedifferentiated liposarcoma*		
Arg1	Arginase	133.05
Crabp2	Cellular retinoic acid binding protein 2	18.87
Scin	Scinderin	15.47
Mgam	Maltase-glucoamylase (alpha-glucosidase)	8.11
Irx5	Iroquois homeobox 5	7.72
Ncam1	Neural cell adhesion molecule 1	6.55
Tnfsf9	Tumor necrosis factor (ligand) superfamily, member 9	4.99
Cldn10	Claudin 10	4.47
Ldlr	Low-density lipoprotein receptor	3.29
Meis2	Meis homeobox 2	3.28
Vegfa	Vascular endothelial growth factor A	3.14
Got1	Glutamic-oxaloacetic transaminase 1	3.05
Nkd2^#^	Naked cuticle homolog 2	2.50
Aurka^#^	Aurora kinase A	2.30

Genes of interest include those that regulate lipid, fatty acid and carbohydrate metabolism, cell proliferation, apoptosis, transcription, insulin-like growth factor pathway, Wnt signaling and obesity

*P-value ≤0.02. ^#^Fold change between 2 and 3

## Abbreviations

LPS: liposarcoma
WDLPS: well-differentiated liposarcoma
DDLPS: dedifferentiated liposarcoma
MLPS: myxoid/round cell liposarcoma
PLPS: pleomorphic liposarcoma
MSCs: mesenchymal stem cells
IHC: immunohistochemistry
LPL: lipoprotein lipase
HGF: hepatocyte growth factor
*MDM2*: mouse double minute 2 homolog
*p53*: tumor protein p53
*Pten*: phosphatase and tensin homolog
PI3K: phosphatidylinositol-3 kinase
AKT: protein kinase B
mTOR: mammalian target of rapamycin
CDK: cyclin-dependent kinases
CDK4: cyclin-dependent kinase 4
CDK6: cyclin-dependent kinase 6
C/EBP delta: CCAAT/enhancer-binding protein delta
*Fabp4*: fatty acid binding protein 4, adipocyte
*Lpl*: lipoprotein lipase
*Pgm5*: phosphoglucomutase 5
*Ldlr*: low-density lipoprotein receptor
*Got1*: glutamic-oxaloacetic transaminase 1
*Ech1*: enoyl CoA hydratase 1
*Igf2*: insulin-like growth factor 2
*Igf1*: insulin-like growth factor 1
*Foxo1*: forkhead box O1
*Foxc2*: forkhead box C2
*Sox6*: SRY (sex determining region Y)-box 6
*Pparg*: peroxisome proliferator-activated receptor gamma
*Irx5*: iroquois homeobox 5
*Meis2*: meis homeobox 2
*Ebf1*: early B-cell factor 1
*Sox17*: SRY (sex determining region Y)-Box 17
*Lep*: leptin (murine obesity homolog)
*Ntrk2*: neurotrophic tyrosine kinase, receptor, type 2
*Nkd2*: naked cuticle homolog 2
*Fzd4*: frizzled class receptor 4
Vegfa: vascular endothelial growth factor A
*G0s2*: G0/G1 switch 2
*Got1*: glutamic-oxaloacetic transaminase 1
*Aurka*: aurora Kinase A
DNMT3A: DNA (cytosine-5)-methyltransferase 3A
SUV39H1: suppressor of variegation 3–9 homolog 1
CREB: cAMP response element-binding protein
