# Supplementary figures and images for: Involvement of tumor suppressors PTEN and p53 in the formation of multiple subtypes of liposarcoma
Source: Cell Death Differ. 2015 Mar 27;22(11):1785–91. doi: 10.1038/cdd.2015.27 (PMC4648325; doi:10.1038/cdd.2015.27)

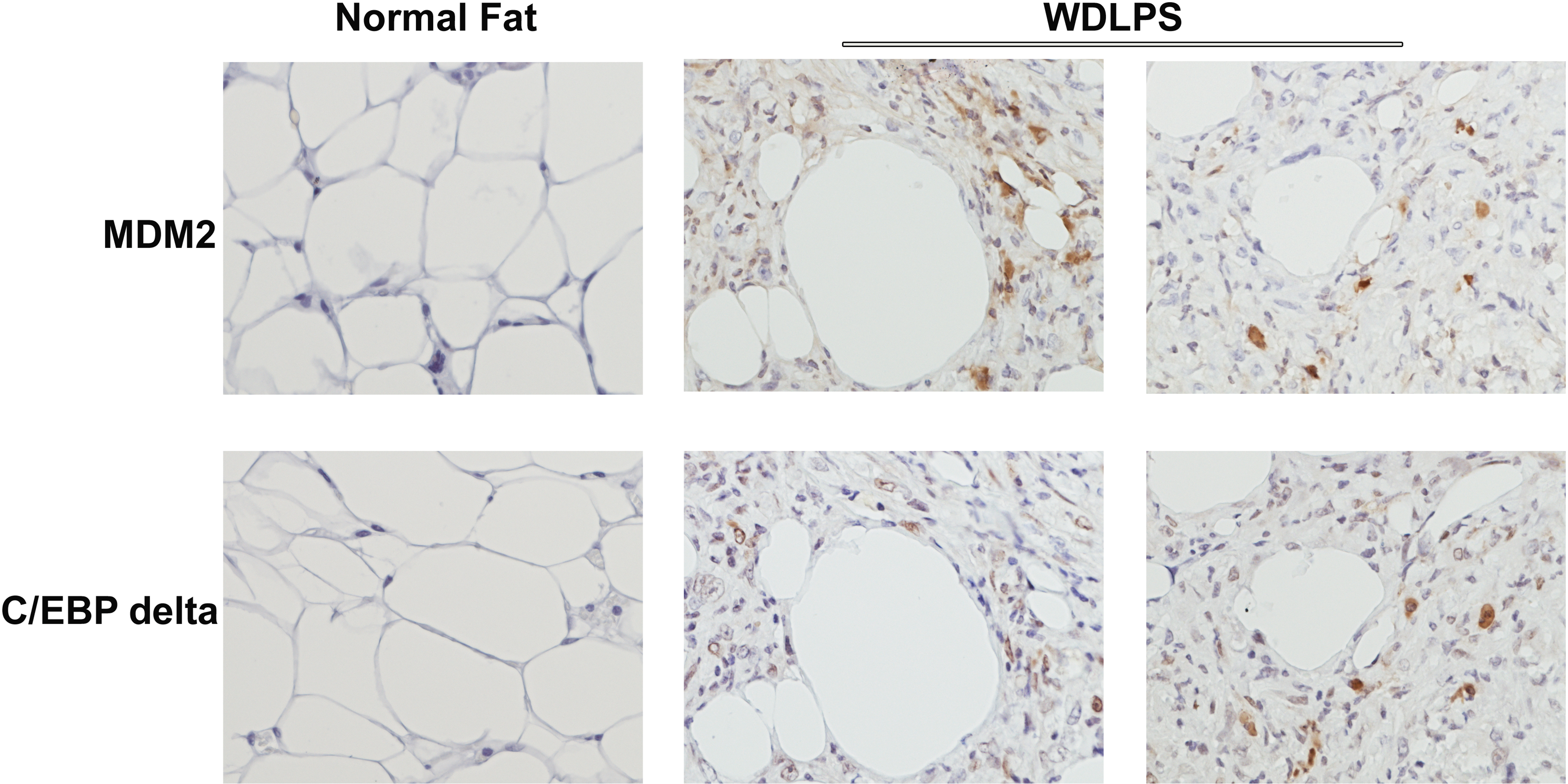

Supplement: Supplementary Figure 1 [file cdd201527x1.tif]
