# Supplementary material for: Involvement of tumor suppressors PTEN and p53 in the formation of multiple subtypes of liposarcoma
Source: Cell Death Differ. 2015 Mar 27;22(11):1785–91. doi: 10.1038/cdd.2015.27 (PMC4648325; doi:10.1038/cdd.2015.27)
Supplement: Supplementary Information [file cdd201527x3.doc]

Supplementary Figure 1

**Immunohistochemical analysis of MDM2 and C/EBP delta in the WDLPS subtype.**

Serial liposarcoma tumor sections show similar patterns of expression between MDM2 protein and C/EBP delta. Normal fat sections were used as negative controls for staining.

Supplementary File 2

**Top differentially expressed genes between DDLPS and WDLPS.**

The file contains the top differentially expressed genes that were used to generate the Heat Map between DDLPS and WDLPS tumors.
